# Discordance of Tuberculin Skin Test and Interferon Gamma Release Assay in Recently Exposed Household Contacts of Pulmonary TB Cases in Brazil

**DOI:** 10.1371/journal.pone.0096564

**Published:** 2014-05-12

**Authors:** Rodrigo Ribeiro-Rodrigues, Soyeon Kim, Flávia Dias Coelho da Silva, Aleksandra Uzelac, Lauren Collins, Moíses Palaci, David Alland, Reynaldo Dietze, Jerrold J. Ellner, Edward Jones-López, Padmini Salgame

**Affiliations:** 1 Cellular and Molecular Immunology Laboratory, Núcleo de Doenças Infecciosas, UFES, Vitória, Brazil; 2 Department of Preventive Medicine and Community Health, New Jersey Medical School, Rutgers Biomedical and Health Sciences, Newark, New Jersey, United States of America; 3 Division of Infectious Diseases, Department of Medicine, New Jersey Medical School, Rutgers Biomedical and Health Sciences, Newark, New Jersey, United States of America; 4 Boston University School of Medicine, Boston, Massachusetts, United States of America; 5 Mycobacteriology Laboratory, Núcleo de Doenças Infecciosas (NDI), Universidade Federal do Espírito Santo (UFES), Vitória, Brazil; 6 Section of Infectious Diseases, Department of Medicine, Boston Medical Center and Boston University School of Medicine, Boston, Massachusetts, United States of America; Emory University, United States of America

## Abstract

Interferon-gamma (IFN-γ) release assays (IGRAs) such as the Quantiferon Gold In-tube test are *in vitro* assays that measure IFN-γ release from T cells in response to *M. tuberculosis* (Mtb)-specific antigens. Unlike the tuberculin skin test (TST), IGRA is specific and able to distinguish Mtb-infection from BCG vaccination. In this study we evaluated the concordance between TST and IGRA and the efficacy of IGRA in diagnosing new Mtb infection in household contacts (HHC) of pulmonary tuberculosis (PTB) cases. A total of 357 HHC of TB cases in Vitória, Brazil were studied. A TST was performed within 2 weeks following enrollment of the HHC and if negative a second TST was performed at 8-12 weeks. HHC were categorized as initially TST positive (TST+), persistently TST negative (TST-), or TST converters (TSTc), the latter representative of new infection. IGRA was performed at 8–12 weeks following enrollment and the test results were positive in 82% of TST+, 48% of TSTc, and 12% of TST-, indicating poor concordance between the two test results among HHC in each category. Evaluating CXCL10 levels in a subset of IGRA supernatants or lowering the IGRA cutoff value to define a positive test increased agreement between TST and IGRA test results. However, ROC curves demonstrated that this resulted in a trade-off between sensitivity and specificity of IGRA with respect to TST. Together, the findings suggest that until the basis for the discordance between TST and IGRA is fully understood, it may be necessary to utilize both tests to diagnose new Mtb infection in recently exposed HHC. Operationally, in IGRA negative HHC, it may be useful to employ a lower cutoff value for IGRA to allow closer monitoring for potential conversion.

## Introduction

Tuberculosis (TB), caused by *Mycobacterium tuberculosis* (Mtb), is still a major human health problem, especially in developing countries. The World Health Organization estimated that there were 8.8 million new cases of TB (12% co-infected with HIV) and 1.34 million people died from TB in 2012 [1]. In most individuals exposed to Mtb, the infection is contained by the host immune response and remains latent. In latent tuberculosis infection (LTBI), the mycobacteria persist and can reactivate at a later time to cause active disease in about 10% of those infected over a lifetime (HIV uninfected). It has been estimated that one-third of the world's population has LTBI, serving as a vast reservoir from which new cases can emerge; hampering TB eradication. Therefore, a major challenge in TB control is to be able to diagnose, predict, and treat those with LTBI before they develop active disease. The diagnosis of Mtb infection is complicated by the lack of a practical gold standard test. In order to assess the predictive utility of diagnostic tests for LTBI, long-term follow-up of individuals from infection to disease is required.

Until recently, the tuberculin skin test (TST) and chest X-ray examination have been the main diagnostic tools used for TB contact investigations. However, chest X-ray examination is only done if LTBI is present in an individual. For close to a century, TST was the only option for the diagnosis of LTBI. TST is based on *in vivo* cell-mediated immune response to Mtb antigens after intra-dermal injection of tuberculin purified protein derivative (PPD), a complex mixture of proteins [2] derived from a human strain of Mtb. A positive TST result indicates that an individual is infected with Mtb, but does not differentiate between latent and active disease [3]–[5]. TST values of ≥10 mm are interpreted as positive in most immunocompetent individuals [6]. However, false-positive TST may occur after infection with environmental nontuberculous mycobacteria that share common antigens with Mtb or after Bacille Calmette-Guérin (BCG) vaccination [6]–[8]. False-negative TST may occur in the presence of HIV infection and other immunosuppressive states or after recent vaccination with live-attenuated viral vaccines [9]. It is common knowledge that errors introduced by misplacing and/or misreading the TST may adversely affect accuracy [10]. False positive TST are also problematic in BCG vaccinated infants, if tested at age ≤10 years. [11]. Therefore, the utility of TST in the identification of LTBI has been challenging in child contacts in countries where BCG vaccination is widely administered in infancy, such as in Brazil. False positive TST remain problematic through adulthood in individuals vaccinated after the first year of life [11].

Access to *in vitro* methods measuring specific cellular immune responses to Mtb offers potential advantages and may circumvent some limitations presented by TST. Identification of the highly specific RD1 region in Mtb genome has enabled the development of new diagnostic tests for Mtb infection. Efforts have concentrated on methods for detecting IFN-γ, a key Th1 cell-associated cytokine necessary for effective host defense against Mtb (reviewed in [12]). Interferon-gamma (IFN-γ) release assays (IGRAs) detect IFN-γ release from lymphocytes after *in vitro* incubation of whole blood with Mtb antigens, such as early secretory antigenic target-6 (ESAT-6) and culture filtrate protein 10 kD (CFP-10). IGRAs offer enhanced specificity by measuring IFN-γ production in response to antigens that are specific to Mtb and, therefore, not influenced by cross-reactivity to *M. bovis* BCG and most non-tuberculous mycobacteria (NTM) [13]. The commercially available ELISA-based QuantiFERON TB Gold IN-TUBE, which includes the TB 7.7 (p4) as an additional antigen, is an example of such an *in vitro* IGRA test.

HHC of pulmonary TB patients are at highest risk of Mtb infection and progression to TB disease [14]. Identification of infected HHC is therefore critical for TB control. Although IGRAs are more specific than TST, serial studies have shown frequent reversions and conversions [15]. In a recent study this temporal change in IGRA was attributed to the variability inherent to the test rather than driven by host and bacterial factors [16]. Only a small number of studies have performed both TST and IGRA to determine the prevalence of LTBI among close contacts of index TB cases [17]–[21] and several of these studies reported discordancy between IGRA and TST [19]–[21]. Indeed, findings from one study indicate that in endemic countries, TST results may be more informative than IGRA to initiate LTBI treatment [19]. Therefore, a side-by-side comparison of TST and IGRA in recently exposed HHC is essential in determining the utility of IGRA in accurately identifying latent infection in a high-risk population, such as Brazil [22], where routine contact tracing programs are in place. The aim of this study was to determine the performance of IGRA compared to TST in recently exposed HHC as part of a larger *International Collaboration in Infectious Diseases Research* (ICIDR) study designed to investigate the extent of Mtb transmission in HHC exposed to an index case (IC) with infectious pulmonary TB (PTB).

## Subjects and Methods

### Study population

The ICIDR cohort began enrollment on March 13, 2008. Adult culture confirmed, AFB smear ≥2+ TB patients were enrolled as index cases; within two weeks of enrollment, their HHC were evaluated for Mtb infection and disease. Only HHC of index cases who fulfilled the following inclusion criteria were enrolled: 1) age ≥18 years with cough ≥3 weeks; 2) new TB episode with ≥1 sputum specimen that was AFB ≥2+ with subsequent growth of *M. tuberculosis* in culture, and; 3) living with ≥3 persons fulfilling the study definition of a contact. We excluded HIV-infected patients (or those who refused HIV testing), patients with a previous history of TB treatment, and patients who were too ill to consent, unable to understand, or comply with, the study protocol. A household contact was defined as an individual of any age fulfilling at least one of the following criteria of close contact with the index TB cases for ≥3 months before enrollment: 1) person sleeping under the same roof ≥5 days/week; 2) person sharing meals ≥5 days/week; 3) person watching TV nights or weekends; and, 4) other significant contact. Primary or secondary prophylaxis with isoniazid was offered to participants meeting guidelines of the Brazilian TB Control Program [23]. We obtained written informed consent and assent in Portuguese in accordance with age-specific ethical guidelines from participating institutions.

Only those index cases and their HHC enrolled by March 19, 2010 were included in this substudy. The following parameters were evaluated in HHC: a) presence of TB disease symptoms; b) TST result; and c) chest X-ray. Eight weeks later, HHC had: a) repeat TST (if 1^st^ <10 mm); b) IGRA testing using QuantiFERON TB-Gold In-Tube; and c) evaluation of the household environment. Blood samples for IGRA were drawn at the second visit and obtained prior to application of a second TST, in those initially TST negative.

### TST

**Figure 1:**
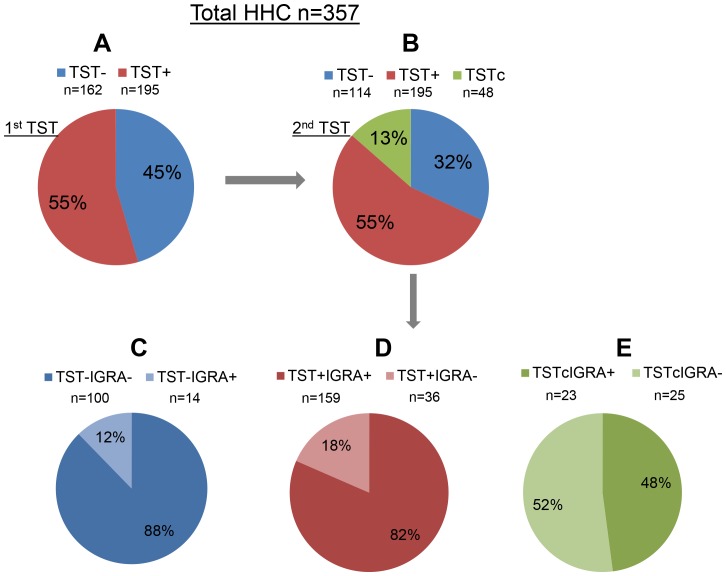
Pie charts show the number and percentage of HHC in each category. IGRA responses were analyzed in the three HHC subgroups (TST-, TST+ and TSTc) and the breakdown is diagrammatically represented.

The initial TST involved an intra-dermal injection of 0.1 mL (2 tuberculin units) of the PPD RT23 (Statens Serum Institute, Copenhagen, Denmark) into the anterior surface of the forearm with a standard tuberculin syringe. Reactions were evaluated 72 to 96 hours after the injection by two expert readers who are certified and have a concordance rate ≥95% with the local TST reference reader. A positive result on the TST was defined as an induration of ≥10 mm in the transverse diameter using the ball pen method. If the first TST result was <10 mm, then a second TST was performed 8–12 weeks following enrollment (Figure 1). TST conversion between 1^st^ and 2^nd^ TST readings was defined as those with initial TST <10 mm and 2^nd^ TST ≥10 mm. We also utilized a second definition of TST conversion (Menzies Criterion) that additionally required the PPD induration to increase by at least 6 mm.

### IGRA using QuantiFERON TB-Gold In-Tube

Blood samples for IGRA testing were collected by venipuncture prior to intradermal injection of PPD using vials provided by the manufacturer. The blood samples were transported to the Núcleo de Doenças Infecciosas (NDI-UFES), at the Federal University of Espirito Santo, in Vitória, Espirito Santo state, Brazil, and used for whole-blood IFN-γ release assay (QuantiFERON TB Gold IN-TUBE, Qiagen, Valencia, CA, USA) according to the manufacturer's instructions. For the whole-blood stimulation step, 1 ml aliquots of whole blood were incubated as follows: a) nil control (saline); b) MTB antigens (early secretory antigenic target 6 [ESAT-6], culture filtrate protein 10 [CFP-10], and TB7.7 (p4)); and c) positive control (mitogen). Following stimulation, IFN-γ levels in whole blood supernatants were quantified by ELISA. A positive result was defined as [(MTB antigen-stimulated IFN-γ level) minus (nil-stimulated IFN-γ level)] ≥0.35 IU/ml. This will be referred to as (TB Ag-Nil), hereafter. TST and IFN-γ assay readings were performed independently, without knowledge of the outcome of the other.

### CXCL10 quantitation in IGRA supernatants

CXCL10 ELISA on the IGRA supernatants was performed using the Meso Scale Discovery (MSD) platform (Meso Scale Discovery, Rockville, MD, USA).

### Data analysis

A generalized estimating equation (GEE) approach was used to estimate confidence intervals for the percentage at or above IGRA cutoffs among TST-positive (TST+) and among TST converters (TSTc), and percentage below the cutoff among TST-negative (TST-), adjusted for clustering within a household. When defining a positive test using a combination of IGRA or CXCL10 values, the percentages were estimated using the inverse probability of being included in the CXCL10 sample as weights in GEE models. Non-parametric ROC curves were estimated and plotted using the pROC (http://cran.r-project.org/) (pROC: an open-source package for R and S+ to analyze and compare ROC curves [24]. For all other analyses SAS 9.2 software (SAS Institute Inc., Cary, NC, USA) was used.

### Ethics Statement

The study was approved by Ethics on Human Research Commitee-Health Sciences Campus - Universidade Federal do Espírito Santo, Conselho Nacional de Etica em Pesquisa (Brazilian National Council On Research Ethics), Boston University Medical Campus Institutional Review Board and RBHS Institutional Review Board. We obtained written informed consent and assent in Portuguese in accordance with age-specific ethical guidelines from participating institutions. Written parental consent was obtained for children <7 years and written assent and written parental consent were obtained for children between 7–17 years. This consent procedure was approved by IRBs of all participating institutions.

## Results

### Demographic characteristics of index cases (IC) and household contacts (HHC)

Of the 467 HHC enrolled in the ICIDR study by the cutoff date, 110 participants could not be included in the analysis because: a) one had active TB disease; b) four had no TST and IGRA testing; c) nine had no TST testing; d) one had a week 8–12 TST but no initial TST; e) twenty-three were TST negative at week 1–2 but had no repeat TST at week 8–12; f) seventy-one had no IGRA testing; and g) one had an indeterminate IGRA result. Therefore, a total of 357 HHC distributed over 76 households were included in this study. For the IC, the median age was 33 years (range: 18 to 81 years), 39% were female and the median body mass index (BMI) was 18.0 kg/m^2^. For HHC, the median age was 20 years (range: 6 months to 87 years), 59.4% were female, and a BCG scar was reported in 77.3%. The observed median BMI among HHC aged 20+ years was 23.2 kg/m^2^

### TST and IGRA Results

Considering a TST induration ≥10 mm as TST positive, 195 (55%) out of the 357 HHC were positive at the 1^st^ TST [(TST+), Figure 1A]. After a second TST test performed 8–12 weeks later on those negative at the first reading, 48 (13%) HHC became newly TST positive [(TSTc), Figure 1B] and a total of 243 out of the 357 HHC were TST positive increasing the frequency from 55% to 68% (Figure 1B). The remaining 114 (32%) HHC were TST negative at both at 1^st^ and 2^nd^ testing [(TST-), Figure 1B].

IGRA responses were analyzed in the three HHC subgroups (TST-, TST+ and TSTc) and the breakdown shown in Table 1 and diagrammatically represented in Figure 1 is as follows: i) Of the 114 TST- HHC, 100 (88%) were IGRA negative (TST-IGRA-); and 14 (12%) were IGRA positive [(TST-IGRA+) Figure 1C]; ii) Of the 195 TST+ HHC, 159 (82%) were also IGRA positive (TST+IGRA+) and the remaining 36 (18%) were IGRA negative [(TST+IGRA-) Figure 1D]; iii) Of the 48 TSTc HHC, 23 (48%) were also IGRA positive (TSTcIGRA+) and the remaining 25 (52%) were IGRA- [(TSTcIGRA-) Figure 1E]. Taken together, a total of 75 (21%) HHC showed discordance between TST and IGRA [Kappa = 0.57 (95% confidence interval (CI): 0.48, 0.65)], with 61 TST+IGRA- and 14 TST-IGRA+, a ratio of 4.4 (95% confidence interval (CI): 2.4, 7.8), indicating that, when tests were discordant, TST was more likely to be the test that was positive (p<0.001).

**Table 1:** Characteristics of Household Contacts.

Characteristic	Total	TST-/IGRA-	TST-/IGRA+	TSTc/IGRA-	TSTc/IGRA+	TST+/IGRA-	TST+/IGRA+
	(n = 357)	(n = 100)	(n = 14)	(n = 25)	(n = 23)	(n = 36)	(n = 159)
Female gender: n (%)	212 (59.4)	57 (57.0)	8 (57.1)	18 (72.0)	18 (78.3)	22 (61.1)	89 (56.0)
Age (years): median (range)	20 (0.5,87)	14 (0.5,78)	12.5 (5,72)	26 (1,60)	21 (3,72)	29.5 (4,75)	20 (1,87)
History of TB diagnosis: n (%)	7 (2.0)	0 (0.0)	1 (7.1)	0 (0.0)	0 (0.0)	0 (0.0)	6 (3.8)
BCG scar present: n (%)							
yes	276 (77.3)	80 (80.0)	11 (78.6)	23 (92.0)	16 (69.6)	26 (72.2)	120 (75.5)
no	68 (19.0)	15 (15.0)	3 (21.4)	2 (8.0)	5 (21.7)	9 (25.0)	34 (21.4)
uncertain	13 (3.6)	5 (5.0)	0 (0.0)	0 (0.0)	2 (8.7)	1 (2.8)	5 (3.1)
Average hours/day exposure to index case in past 3 months: n (%)							
<7 Hrs/Day	128 (35.9)	46 (46.0)	4 (28.6)	14 (56.0)	3 (13.0)	24 (66.7)	37 (23.3)
7–12 Hrs/Day	94 (26.3)	29 (29.0)	5 (35.7)	5 (20.0)	7 (30.4)	5 (13.9)	43 (27.0)
13–18 Hrs/Day	83 (23.2)	20 (20.0)	4 (28.6)	2 (8.0)	6 (26.1)	4 (11.1)	47 (29.6)
>18 Hrs/Day	52 (14.6)	5 (5.0)	1 (7.1)	4 (16.0)	7 (30.4)	3 (8.3)	32 (20.1)
TST PPD induration at entry: median (range)	11 (0, 30)	0(0, 8)	0 (0, 6)	5 (0, 9)	0 (0, 9)	14 (10, 21)	16 (10, 30)
TST PPD induration by week 8–12*: median (range)	14 (0, 30)	0(0, 9)	0 (0, 9)	14(10, 22)	18(10, 25)	14(10, 21)	16(10, 30)
Body mass index (kg/m^2^) (≥age 20 years): n	186	44	6	17	12	27	80
median (range)	23(15, 36)	25(15, 36)	20(17, 33)	24(16, 29)	23(17, 31)	24(16, 29)	22(15, 34)
TB ag-Nil (IU/ml): median (range)	0.6(−4.2, 42.9)	0.0(−0.6, 0.2)	1.0(0.5, 9.9)	0.0(−2.7, 0.3)	10.0(0.5, 21.1)	0.1(−4.2, 0.3)	5.4(0.4, 42.9)

*Since those with PPD induration at entry of ≥10 mm did not have repeat testing, the induration for that group is from the entry TST but for those with entry TST<10 mm this variable uses the week 8–12 value.

Although there is no established gold standard for LTBI, our study treated TST as a surrogate reference standard for the purpose of elucidating the operational characteristics of IGRA with respect to TST. Our study design allowed us to compare TST converters to those TST positive at first evaluation. IGRA only identified 52% of converters (Figure 1E). Whereas those HHC with 1^st^ TST positive may be a heterogeneous group, potentially including individuals with longstanding Mtb infection (unrelated to the index case exposure) and/or BCG cross-reactive individuals in addition to recent infection (unobserved TST converters), IGRA identified 82% of these individuals (Figure 1D), a significantly greater percentage compared to TST converters (p<0.001).

Analysis of the presence of BCG scars showed that the proportion of HHC with a scar was similar in the 6 sub-groups indicating that it was unlikely that the discordance was influenced by BCG vaccination (Table 1). In order to exclude the possibility that enhanced TST positivity could be related to BCG cross-reactivity, we compared TST and IGRA in only those HHC whose 1^st^ TST was negative (TST- and TSTc). Of the 162 HHC whose 1^st^ TST was negative (includes TST- and TSTc), 39 (24%) were discordant for TST and IGRA, (Kappa = 0.38 (95% CI: 0.22, 0.54), with the ratio of TST converters/IGRA negative (n = 25) to TST negative (second test also)/IGRA positive (n = 14) being 1.8 (95% CI: 0.9, 3.4). This indicates a reduced but marginally significant (p = 0.078) greater estimated proportion of Mtb infection as defined by TST compared to IGRA even when restricting our sample to a population where the possibility of BCG cross-reactivity was minimized. Interestingly, with regards to exposure to the index case (measured as average hrs/day exposure to the index case over a 3 month period), the discordant groups (TSTc/IGRA- and TST+/IGRA-) had a less intense exposure compared to the concordant positive groups (TSTc/IGRA+ and TST+/IGRA+) (trend test p<0.001) (Table 1).

**Figure 2:**
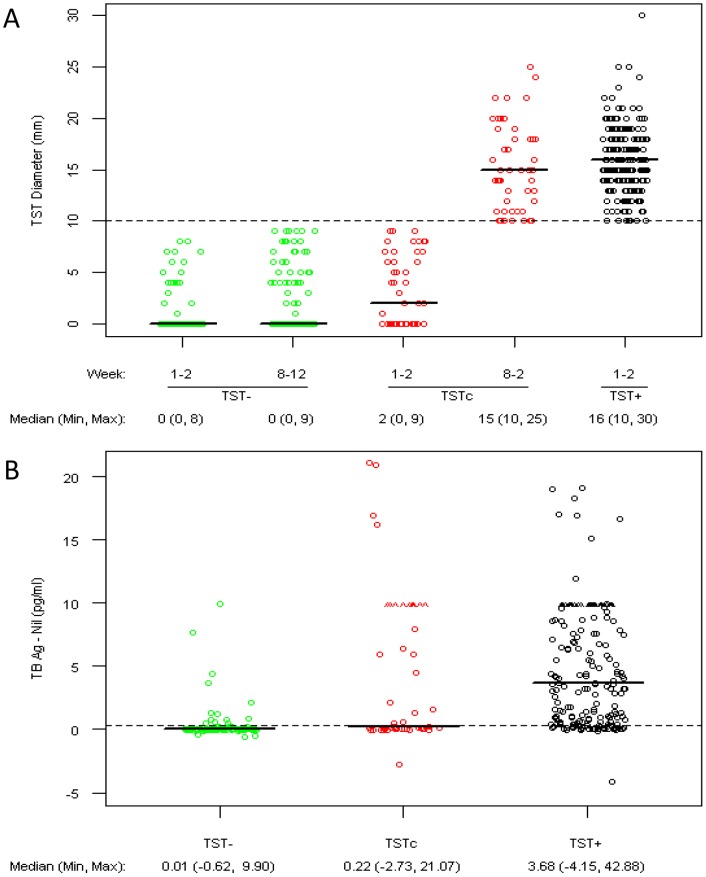
Comparison of the magnitude of TST and IGRA responses. A) PPD Diameter for 1^st^ and 2^nd^ TST in TST-negative (TST-), TST-converter (TSTc), and Prevalent TST positive (TST+) groups displayed by PPD testing visit and TST group*. B) IGRA results using Quantiferon Gold In-tube quantitative TB Ag – Nil by TST-, TSTc, and TST+ groups. 2^nd^ TST is only performed when PPD diameter in TST1 is <10 mm. Dashed line in Figure 2A at 10 mm is the cutoff for a positive TST result. ∧ indicates values greater than 10 IU/ml (prior to December 2009 levels ≥10 IU/ml were reported as 10+). A dashed line in Figure 2B is shown at 0.35 IU/ml, the cutoff for a positive IGRA test result.

Comparison of the magnitude of the TST response by PPD diameter in the TSTc at week 8–12 was similar to that of the TST+ group at entry; a median of 15 mm in TSTc compared to 16 mm in TST+ (p = 0.15) (Figure 2A). However, comparison of the magnitude of the IGRA responses in the three groups showed that in the TSTc, the majority of the responses clustered around the cut-off value with a median TB Ag – Nil of 0.22 IU/ml (Figure 2B). Furthermore, the median TB Ag – Nil value in the TSTc group, though statistically different (p<0.001), was only 0.21 IU/ml higher than that of the TST- group and below the 0.35 IU/ml positivity level recommended by the manufacturer (Figure 2B). In contrast, the TSTc had a median TB Ag – Nil value (2.46 IU/ml) lower than that of the TST+ group, a substantial difference which was also statistically significant (p = 0.003). Together, these data indicate that there is a high level of IGRA discordance in the TSTc group and that the majority of the discordancy is due to IGRA results that are close to the cutoff value.

### Analysis of CXCL10 levels in IGRA supernatants in a subset of HHC

CXCL10, an IFN-γ inducible chemokine involved in Th1 cell migration to inflammatory sites [25] has been studied as an alternative marker for discriminating between LTBI and active TB [26]–[29]. Next, we determined whether discordance between TST and IGRA would be reduced by additionally determining CXCL10 levels in IGRA supernatants. Supernatants from 150 participants were sampled, with those that were discordant IGRA negative, i.e., TST-positive/IGRA-negative or TST converters/IGRA-negative, oversampled. As shown in Figure S1, CXCL10 levels from both unstimulated and stimulated IGRA supernatants were significantly increased in subjects that were IGRA positive when compared to their negative counterparts. Since the levels of CXCL10 in the TSTc/IGRA- and TST+/IGRA- groups were similar to that in TST-/IGRA- group, the addition of CXCL10 measurement did not improve IGRA concordance with TST.

### Improving IGRA and TST concordance by changing IGRA cutoffs

**Figure 3:**
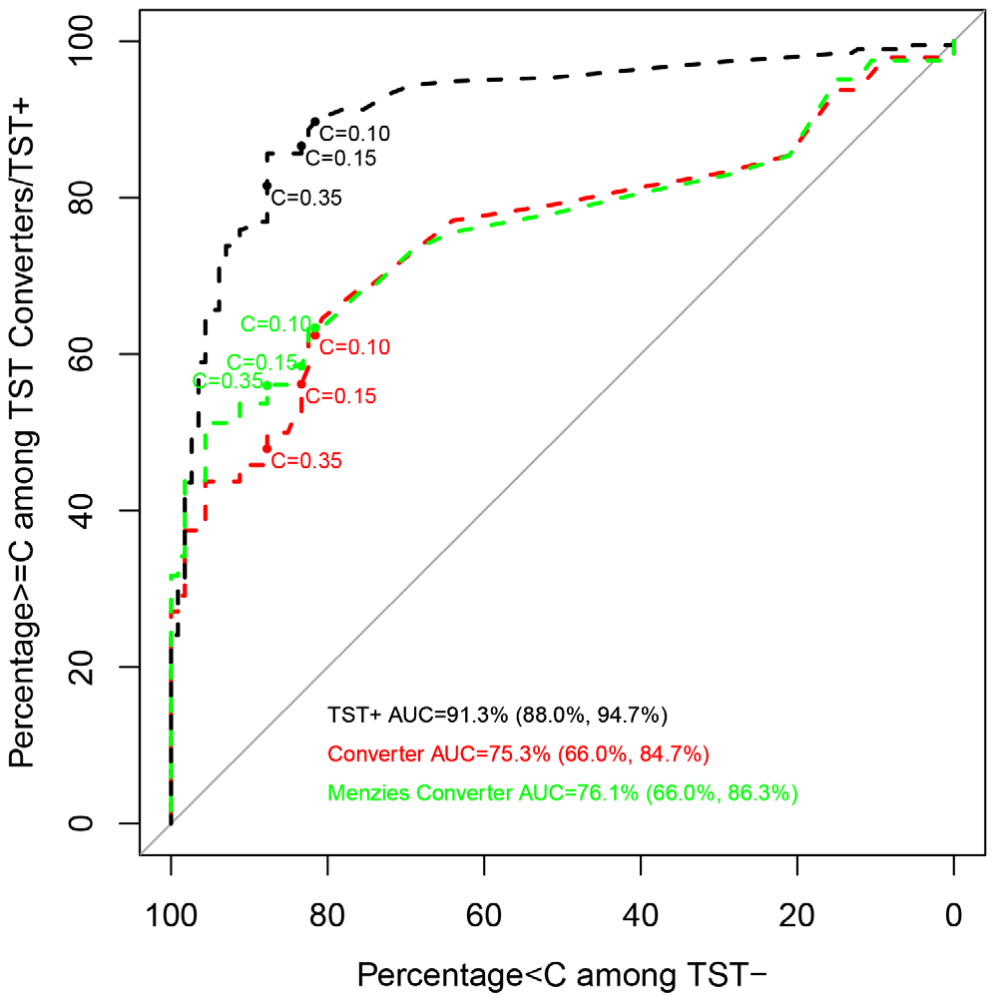
ROC curves for TBag − Nil. Statistics displayed on the curves are C = *X*: *Y*, *Z*, where *X* is the TB Ag − Nil (IU/ml) cutoff value for which IGRA would be declared positive. *Y* is Percentage of TST-negative (Specificity) that is below the cutoff and *Z* are the Percentage of TST-positive or TST converters at or above the cutoff (Sensitivity).

Receiver operating characteristic (ROC) curves were generated to show the trade-off of sensitivity and specificity by varying the quantitative IGRA cutoff defining a positive result, i.e., the TB Ag – Nil values when “true positive” is defined as HHC whose TST converted between 1^st^ to 2^nd^ tests (TSTc) and “true negative” is defined as HHC whose baseline and follow-up TST were both negative (TST-). A second curve was superimposed which uses the same “true negative” definition but defined “true positive” as HHC with an initial TST positive result (TST+). The ROC highlights estimates corresponding to the standard cutoff value (TB Ag – Nil  = 0.35 IU/ml) and two more liberal cutoff values (TB Ag – Nil  = 0.10 and 0.15 IU/ml) chosen by inspection *post-hoc* to afford favorable trade-offs between sensitivity and specificity. As shown in Figure 3, specificity and sensitivity obtained using the standard cutoff value (TB Ag – Nil  = 0.35 IU/ml) were 87.7% and 47.9%, respectively. When a cutoff value of 0.15 IU/ml was considered, sensitivity increased to 56.3%, and when the cutoff was 0.10 IU/ml sensitivity further increased to 62.5%. Although sensitivity was improved by the use of alternate cutoff values, specificity diminished considerably to 83.3% and 81.6%, for cutoffs of 0.15 and 0.10 IU/ml, respectively (Figure 3, Table 2). For virtually the entire range of cutoff values for TB Ag – Nil, the ROC curve for TST+ HHC lays above that for TSTc, indicating better sensitivity for any given specificity (Figure 3). This result indicates that IGRA is less useful for distinguishing TST negative individuals from TST converters as compared to TST negative from prevalent TST positive, regardless of what cutoff value was considered.

**Table 2:** Sensitivity and specificity using alternate cutoff values.

Definition of Test+	Statistic	Estimate
TB Ag-Nil≥0.35 IU/ml	Pr(T+|Prevalent TST+)	81.5% (74.1%, 87.2%)
	Pr(T+|TST Converter)	47.9% (34.6%, 61.5%)
	Pr(T+|TST Converter excluding differences<6 mm)	57.9% (40.9%, 73.2%)
	Pr(T-|TST-)	87.7% (93.3%, 78.6%)
TB Ag-Nil≥0.15 IU/ml	Pr(T+|Prevalent TST+)	86.7% (80.2%, 91.2%)
	Pr(T+| TST Converter)	56.3% (41.4%, 70.0%)
	Pr(T+|TST Converter excluding differences<6 mm)	60.5% (43.1%, 75.6%)
	Pr(T-|TST-)	83.3% (90.1%, 73.2%)
TB Ag-Nil≥0.10 IU/ml	Pr(T+|Prevalent TST+)	89.7% (84.1%, 93.6%)
	Pr(T+| TST Converter)	62.5% (47.3%, 75.6%)
	Pr(T+|TST Converter excluding differences<6 mm)	63.2% (44.6%, 78.5%)
	Pr(T-|TST-)	81.6% (88.8%, 71.2%)
TB Ag-Nil≥0.35 IU/ml, CXCL10 Stimulated/Unstimulated Ratio≥7	Pr(T+|Prevalent TST+)	85.3% (74.7%, 91.9%)
	Pr(T+| TST Converter)	61.9% (39.3%, 80.3%)
	Pr(T-|TST-)	78.7% (88.9%, 63.2%)
TB Ag-Nil≥0.35 IU/ml, CXCL10 Stimulated/Unstimulated ≥8	Pr(T+|Prevalent TST+)	84.6% (73.9%, 91.4%)
	Pr(T+| TST Converter)	61.9% (39.3%, 80.3%)
	Pr(T-|TST-)	78.7% (88.9%, 63.2%)
TB Ag-Nil≥0.35 IU/ml, CXCL10 Stimulated/Unstimulated ≥10	Pr(T+|Prevalent TST+)	83.1% (72.5%, 90.2%)
	Pr(T+| TST Converter)	55.0% (30.2%, 77.5%)
	Pr(T-|TST-)	83.2% (91.8%, 68.7%)
TB Ag-Nil≥0.35 IU/ml, CXCL10-stimulated≥8,500 pg/ml	Pr(T+|Prevalent TST+)	86.0% (76.6%, 92.1%)
	Pr(T+| TST Converter)	61.9% (36.1%, 82.4%)
	Pr(T-|TST-)	78.7% (87.8%, 65.6%)
TB Ag-Nil≥0.35 IU/ml, CXCL10-stimulated ≥11,000 pg/ml	Pr(T+|Prevalent TST+)	86.0% (76.6%, 92.1%)
	Pr(T+| TST Converter)	55.0% (31.1%, 76.8%)
	Pr(T-|TST-)	83.2% (91.1%, 70.6%)
TB Ag-Nil≥0.35 IU/ml, CXCL10-stimulated ≥12,000 pg/ml	Pr(T+|Prevalent TST+)	86.0% (76.6%, 92.1%)
	Pr(T+| TST Converter)	51.5% (28.1%, 74.3%)
	Pr(T-|TST-)	85.5% (93.1%, 72.0%)

Identification of prevalent TST+, TST converters, and TST- using i) TB Ag − Nil cutoffs, ii) combinations of TB Ag-Nil and CXCL10 Stimulated levels or CXCL10 Stimulated/Unstimulated ratio and iii) Menzies definition (excludes those with difference in TST<6 mm), and TST- using TB Ag − Nil cutoff OF 0.25 IU/ml. Participants come from 74 (IGRA testing)/46 (IGRA and CXCL10 testing) households. Estimation uses a GEE approach with an independent working correlation matrix to adjust for clustering in households. Models based on the sample that included CXCL10 cutoffs were weighted to reflect the larger study cohort.

Pr(T+|Prevalent TST+) is the percentage test-positive among those who are were TST+ at week 1–2.

Pr(T+|TST Converter) is the percentage test-positive among those who are TST-converters (TST- at week 1–2 and TST+ at week 8–12).

Pr(T+|TST Menzies Converter) is the percentage test-positive among those who are TST-converters (TST- at week 1–2 and TST+ at week 8–12 and change of at least 6 mm).

Pr(T-| TST-) is the percentage test-negative among those who are TST-negative at weeks 1–2 and 8–12.

### Improving IGRA and TST concordance by using Menzies criterion for TST conversion

To determine whether TST conversion was due to boosting, the data were also analyzed using the Menzies criterion which is designed to distinguish between true conversion and boosting [30] in the analysis. Analysis of the data by excluding TSTc who had a difference of <6 mm between the 1^st^ and 2^nd^ TST tests (not meeting the Menzies criterion) showed that the AUC for when all converters were included in the analysis versus converters using the Menzies criterion was similar overall (Figure 3). However, the curves diverged near the standard cutoff (TB Ag – Nil  = 0.35 IU/ml) and the sensitivity increased from 47.9% to 56.1% when using the Menzies criterion for TST conversion (Table 2). Small increases in sensitivity were seen when the two alternate cutoff values for IGRA were used. This result suggests that IGRA sensitivity increases when the TST result is corrected for possible boosting effects but is still less useful at distinguishing TST negative individuals from TST converters and than from TST+.

### Improving IGRA and TST concordance by inclusion of CXCL10 values

In addition to evaluating alternate IGRA cutoff values to improve IGRA and TST concordance, several combinations of IGRA and CXCL10 values were analyzed, using the CXCL10 stimulated/unstimulated ratio or just the stimulated value alone (Table 2). As depicted in Table 2, inclusion of CXCL10 levels to determine test positivity showed that there was an improvement in concordance in both the TST+ and TSTc groups; however, there was also an increase in positivity in the TST- group (Table 2). For example, using a CXCL10-stimulated threshold of 8500 pg/ml for a positive result in addition to those who had TB Ag – Nil >0.35 IU/ml to define a positive test gave sensitivity for detecting TSTc of 61.9%, sensitivity for TST+ of 86.0%, and specificity for TST- of 78.7%. With the addition of CXCL10-stimulated >8500 pg/ml to define a positive test, an additional 14.0% of the TSTc tested positive, but at the cost of an additional 9.0% of TST- HHC testing positive. Using a CXCL10 stimulated/unstimulated ratio cutoff of 8 had largely similar trade-offs. Trade-offs achieved by using CXCL10 to improve sensitivity could be similarly achieved by decreasing the threshold of TB Ag – Nil for a positive test to 0.10 IU/ml (Table 2).

### Differences in IGRA and TST concordance by BCG status

Of the 357 household contacts, BCG scar was present for 276, absent for 68, and missing for 13; the latter were excluded. In the BCG-negative subgroup, a higher percentage was estimated to be positive for TST and IGRA compared to the BCG-positive but these differences were not statistically significant. While we found similar Kappa statistics comparing final TST and IGRA results by BCG status (Kappa = 0.54 and 0.56 for BCG- and BCG+ subgroups, respectively), the percentage of the TSTc with positive IGRA was higher in BCG- subjects than among the BCG+ (71.4% vs 41.0%, respectively); however, this difference was not statistically significant (p = 0.22). The percentage IGRA-negative among TST- was similar between BCG- and BCG+ (83.3% vs 87.9%, respectively, p = 0.62) (Table S1). When we compared the TB Ag – Nil ROC curves and used the area-under-the-curve (AUC) to quantify the trade-off in sensitivity and specificity using TSTc and TST- contacts as the gold standard between BCG- and BCG+ groups, we found a significant difference in the AUC (p = 0.007) (Figure S2), indicating that TB Ag – Nil better separates TSTc from TST- among BCG- compared to BCG+. Without a gold standard for Mtbinfection it is not clear whether the difference in AUC is due to superior identification of infection in the BCG- subgroup by TST, IGRA, or some combination of the two.

## Discussion

Once IGRA became commercially available, numerous studies were conducted to determine the performance of the test, including sensitivity, specificity and accuracy. A systematic review of the literature and meta-analysis of the data therein found that commercial IGRAs had a higher positive and negative predictive value for progression from latent infection to active disease [31]. Findings from another meta-analysis review also showed that the IGRA test had high specificity and was not influenced by BCG vaccination [32]. In Korea, a country of intermediate TB incidence, IGRA correlated significantly better with increased risk of infection among contacts compared with the TST using both 10 mm and 15 mm cutoffs [33]. However, discordant TST and IGRA results [20], [21], [34], concerns regarding the test performance characteristics of IGRA [35]–[39] and inconsistency in IGRA test results [16] have been reported. In the present study we evaluated the performance of a commercial IGRA (QuantiFERON TB Gold IN-TUBE) in comparison to TST as part of a HHC study that we are conducting in Vitória, Brazil. We found 21% discordance between the two tests and surprisingly a greater number of HHC were TST positive and IGRA negative compared to those who were TST negative and IGRA positive.

BCG vaccination when given after the first year of age results in larger and persistent TST reactions while vaccination in infancy has minimal effect on TST, when the TST is performed 10 years or more after BCG vaccination [11]. Since the Brazilian population is BCG vaccinated at birth and the median age of contacts was 20 years, BCG cross-reactivity is unlikely to explain the substantial discordance observed in the TST positive HHC who are IGRA negative. We did not perform separate analysis for study participants ≤5 years of age since only 33 children, including 13 with positive 1^st^ TST and 3 TST converters, were ≤5 years. Therefore in our study we were not able to assess whether BCG status was a contributing factor among younger HHC. However, a study that reported only moderate agreement between IGRA and TST in children with LTBI, found that the discordance was not due to BCG vaccination induced false-positive TST test [34]. The observations that IGRA negativity is present in the TST converter group and that percentage of HHC with BCG scars is similar in the discordant and concordant subgroups further support that the TST and IGRA discordancy is unlikely to be due to cross-reactivity from BCG vaccination. On the other hand, when only defining TST converters as true positives, TST and IGRA concordance was higher in the BCG- group as summarized by the ROC AUC, but these results are still difficult to interpret in the absence of a true gold standard for TB infection. Other studies have also reported that BCG does not influence TST and IGRA test discordance in non-pediatric populations [40], [41].

In a study by Anibarro and colleagues [42] the concordance between TST and IGRA tests in HHC, as well as correlation of both tests with the degree of TB exposure, both at baseline and at 2 months were evaluated. The authors reported that concordance between TST and IGRA, and correlation with intensity of Mtb exposure, both were improved at the 2-month time period. Consistent with this report, we also observed that HHC that were TST positive but IGRA negative had less exposure to the index case compared to the concordant group. These data suggest that following Mtb exposure, IGRA conversion may take longer than TST. Clearly, negative IGRA tests should be interpreted with caution, particularly in a high risk setting given that TST performs rather well in high Mtb burden settings [43].

A study aimed at assessing the discordance between IGRA, T-SPOT.*TB*, and TST for the diagnosis of LTBI in a low prevalence population found that the three tests identified different people as test positive, suggesting that most positives from IGRA, T-SPOT.*TB*, and TST are false-positives in low-prevalence populations [44]. False positive IGRA test also pose a significant challenge with detection of conversion in health care workers (HCW) from low-risk settings [45]. As demonstrated in this retrospective study, 64.8% of the HCW that had converted IGRA showed reversion to negative on a repeat IGRA test. However, if the cutoff values were increased to 5.3 IU/ml from the manufacturer's cutoff value of ≥0.35 IU/ml, then the conversion rates were very similar to the historical conversion rate seen with TST in HCW in the institute where the study was performed [45]. Serial IGRA testing in HCW in Cape Town South Africa showed significant intra-individual variability in the magnitude of the IFN-γ responses [46]. Another study conducted in German HCW also reported within-subject variability of serial IGRA tests, particularly those with values around the cutoff [47]. Significant variability was also observed in IGRA test results upon retesting of the same samples conducted in a low TB incidence setting [16]. A conclusion that was derived from the systematic review of all IGRA studies performed in HCW was that IGRAs are not superior to TST in identifying the incidence of new Mtb infection [48]. These studies show the importance of interpreting both TST and IGRA results in the context of exposure and prevalence. Serial studies in larger cohorts are necessary to determine whether IGRA reversion in an individual is an indication of Mtb infection that is cleared or whether it is truly false positive. In this regard, there is growing consensus that the current IGRA cutoff value requires reevaluation and borderline results need to be interpreted with prudence [16], [49].

Quantitation of additional analytes in IGRA supernatants has been to shown to distinguish between active disease and Mtb infection in children [50]. Inclusion of CXCL10 improved the performance of the IGRA test and helped to discriminate infected from non-infected subjects [51]. In response to Mtb-specific antigens, high-risk children expressed significantly higher levels of CXCL10 and IL-2 than low-risk groups and the levels strongly correlated with Mtb exposure and grade of infectiousness of index cases. The study also found an excellent agreement between CXCL10, IL-2 and IGRA [28]. In the present study, we found that by either lowering the IGRA cutoff value or by including the analysis of CXCL10 in the IGRA supernatants, sensitivity was enhanced but at the cost of compromising the specificity of the IGRA test and that we could obtain similar trade-offs by either strategy. Additional studies are required to determine whether inclusion of the evaluation of other cytokines/chemokines will improve IGRA's sensitivity.

Variability in repeat test results and poor specificity in certain populations where sensitivity to non-tuberculous mycobacteria is widespread [52] prompted modification of the initial 10 mm cutoff value for TST. Interpretation of the TST results now takes into account the clinical setting, with the current recommendation of 15 mm cutoff for low prevalence population and a 5 mm cutoff for high-risk population [53]. Similarly, there is growing consensus that IGRA cutoff of 0.35 IU/ml for distinguishing positive and negative test results should also be reconsidered [54]. Currently, there are a number of different recommendations for IGRA use [55]. Contacts of TB patients, particularly within the one year window following exposure, are at increased risk of developing TB [56]. It is therefore important that proper guidelines for IGRA use and determination of the cutoff value be established in intermediate/high incidence countries by conducting serial IGRAs and side-by-side comparison with TST. Until such data are available, it may be important for screening programs to recommend retesting of individuals who are IGRA negative, particularly those who are recently exposed HHC.

## Supporting Information

Figure S1 **Quantification of CXCL10.** CXCL10 levels in unstimulated and stimulated supernatants from QFT whole blood cell cultures separated by TST and IGRA response categories.
(TIF)

Figure S2 **ROC curves stratified by BCG status.** BCG+: 146 TST+, 39 TST Converters (34 if increase<6 mm excluded), 91 TST-. BCG-: 43 TST+, 7 Converters (5 if increase<6 mm excluded), 18 TST-. Tests comparing AUCs for BCG+ vs BCG- for TST+ p = 0.49, Converter p = 0.007, and Menzies Converter p = 0.001.
(TIF)

Table S1 **Differences in IGRA and TST concordance by BCG status.** BCG status was unknown for n = 13. n/N: number IGRA+/total in category. BCG+: P(TST+) = 52.9%, P(Converter) = 14.1%, P(TST-) = 33.0%; 5 of the 39 converters had TST change<6 mm between screening and weeks 8-12. BCG-: P(TST+) = 63.2%, P(Converter) = 10.3%, P(TST-) = 26.5%; 2 of the 5 converters had TST change<6 mm between screening and weeks 8–12. *P-value obtained from a score test from a Generalized Estimating Equation model.
(DOCX)
