# Supplementary figures and images for: Discordance of Tuberculin Skin Test and Interferon Gamma Release Assay in Recently Exposed Household Contacts of Pulmonary TB Cases in Brazil
Source: PLoS One. 2014 May 12;9(5):e96564. doi: 10.1371/journal.pone.0096564 (PMC4018294; doi:10.1371/journal.pone.0096564)

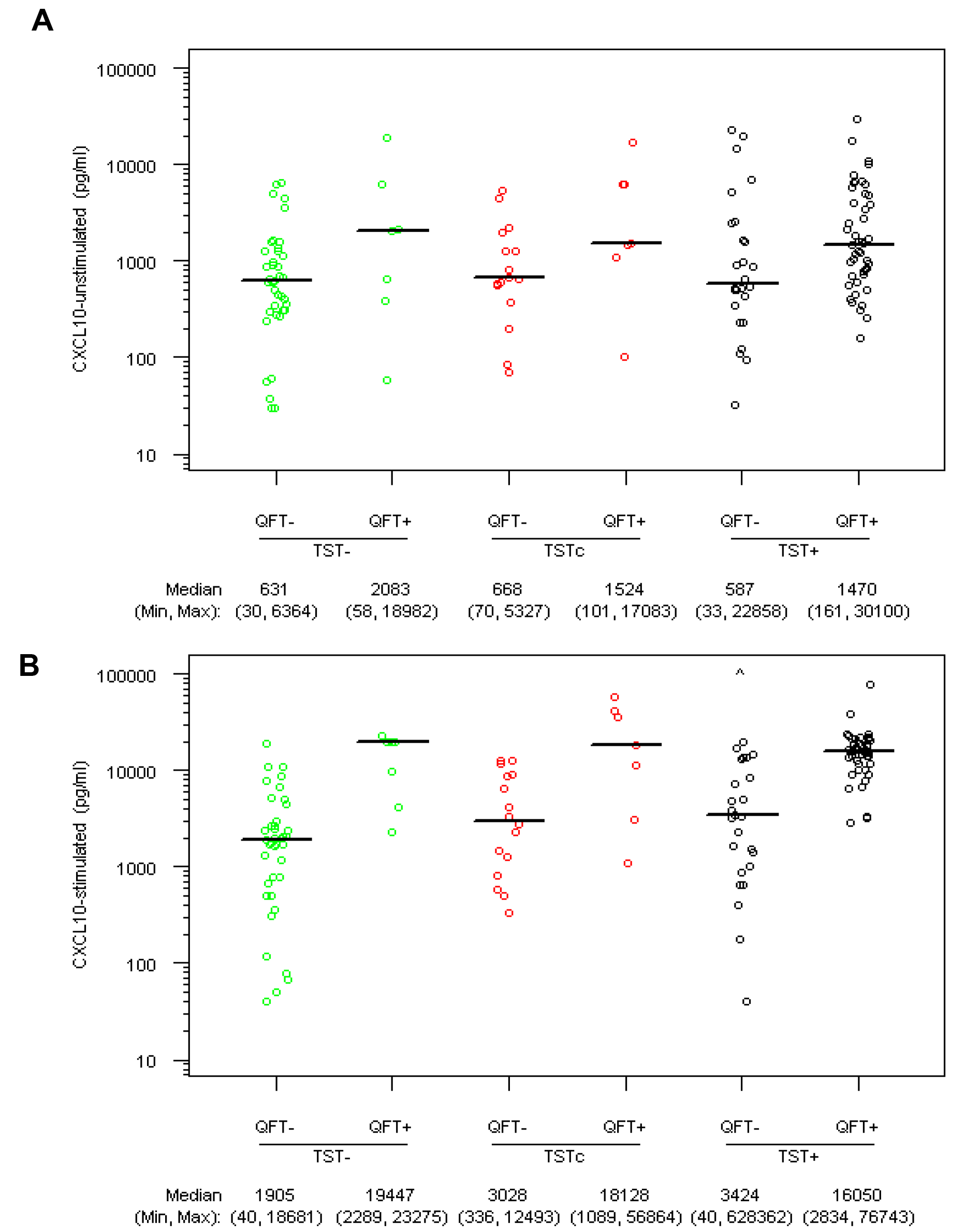

Supplement: Figure S1 — Quantification of CXCL10. CXCL10 levels in unstimulated and stimulated supernatants from QFT whole blood cell cultures separated by TST and IGRA response categories. (TIF) [file pone.0096564.s001.tif]

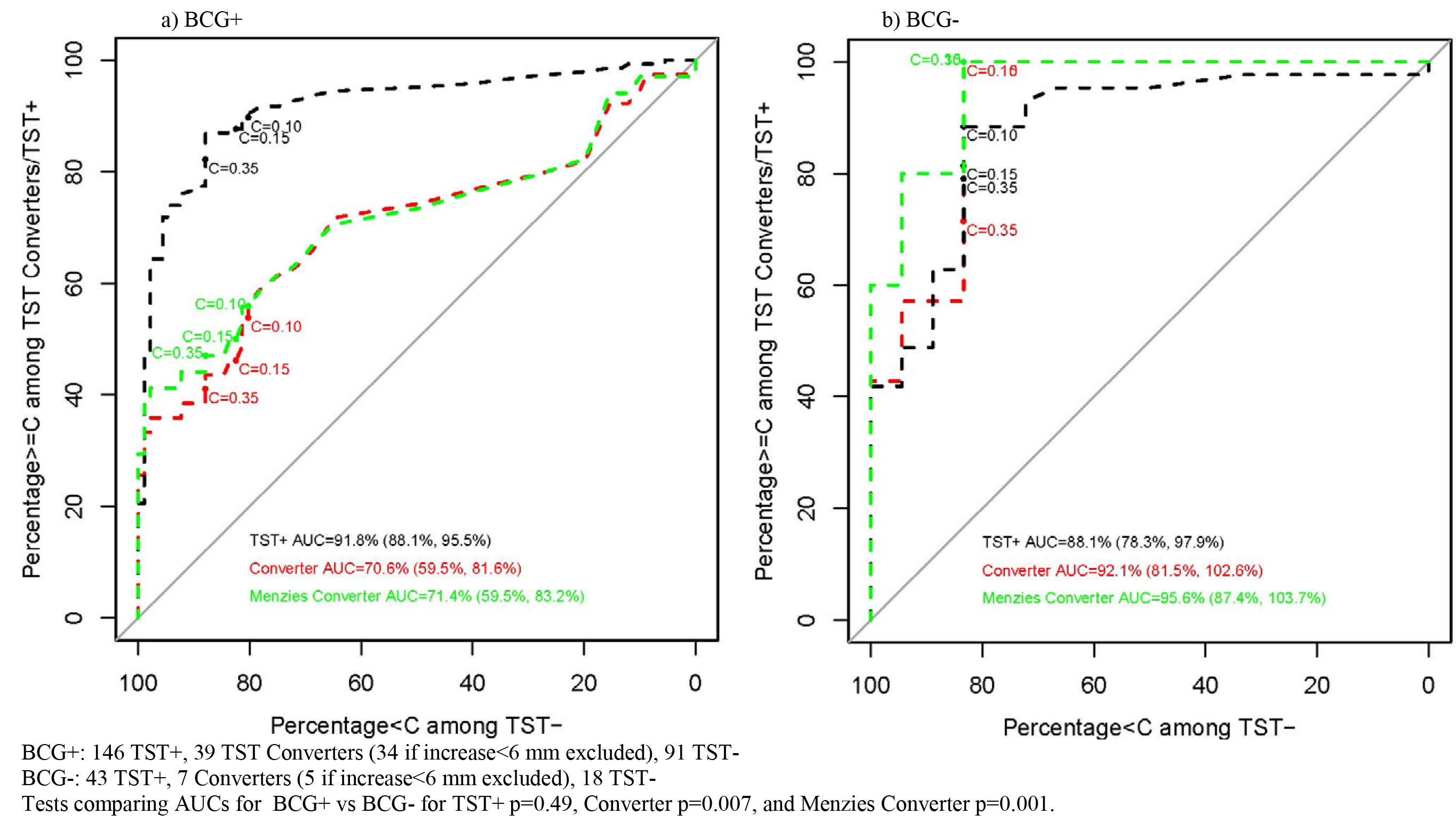

Supplement: Figure S2 — ROC curves stratified by BCG status. BCG+: 146 TST+, 39 TST Converters (34 if increase<6 mm excluded), 91 TST-. BCG-: 43 TST+, 7 Converters (5 if increase<6 mm excluded), 18 TST-. Tests comparing AUCs for BCG+ vs BCG- for TST+ p = 0.49, Converter p = 0.007, and Menzies Converter p = 0.001. (TIF) [file pone.0096564.s002.tif]
