# Supplementary material for: Discordance of Tuberculin Skin Test and Interferon Gamma Release Assay in Recently Exposed Household Contacts of Pulmonary TB Cases in Brazil
Source: PLoS One. 2014 May 12;9(5):e96564. doi: 10.1371/journal.pone.0096564 (PMC4018294; doi:10.1371/journal.pone.0096564)
Supplement: Table S1 — Differences in IGRA and TST concordance by BCG status. BCG status was unknown for n = 13. n/N: number IGRA+/total in category. BCG+: P(TST+) = 52.9%, P(Converter) = 14.1%, P(TST-) = 33.0%; 5 of the 39 converters had TST change<6 mm between screening and weeks 8-12. BCG-: P(TST+) = 63.2%, P(Converter) = 10.3%, P(TST-) = 26.5%; 2 of the 5 converters had TST change<6 mm between screening and weeks 8–12. *P-value obtained from a score test from a Generalized Estimating Equation model. (DOCX) [file pone.0096564.s003.docx]

Table S1: Sensitivity and Specificity of IGRA by BCG status using various test positive cutoffs values

| **Statistic** | **BCG+ (n=276)** | | **BCG- (n=68)** | | **P*** |
| --- | --- | --- | --- | --- | --- |
|  | **n/N** | **Estimate** | **n/N** | **Estimate** |  |
| Pr(IGRA+\|Prevalent TST+) | 120/146 | 82.2% (74.1%, 88.2%) | 34/43 | 79.1% (65.5%, 88.3%) | 0.63 |
| Pr(IGRA+\|TST Converter) | 16/39 | 41.0% (29.5%, 53.7%) | 5/7 | 71.4% (28.9%, 93.9%) | 0.22 |
| Pr(IGRA+\|TST Converter Exclude Diff<6) | 16/34 | 47.1% (33.0%, 61.6%) | 5/5 | 100% (inestimable) | -- |
| Pr(IGRA-\|TST-) | 80/91 | 87.9% (93.8%, 77.6%) | 15/18 | 83.3% (94.5%, 59.1%) | 0.62 |

Figure: ROC curves stratified by BCG


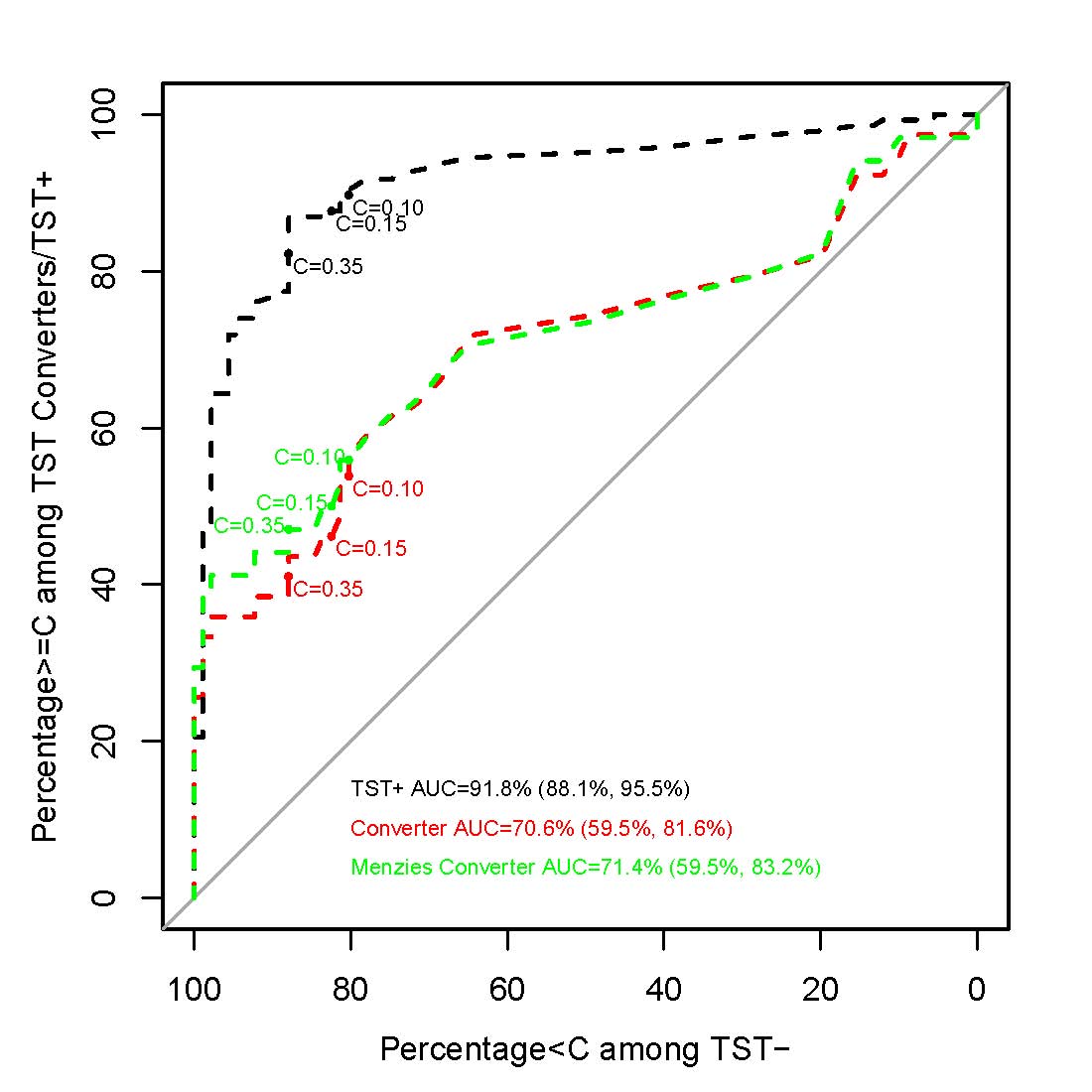
a) BCG+ b) BCG-


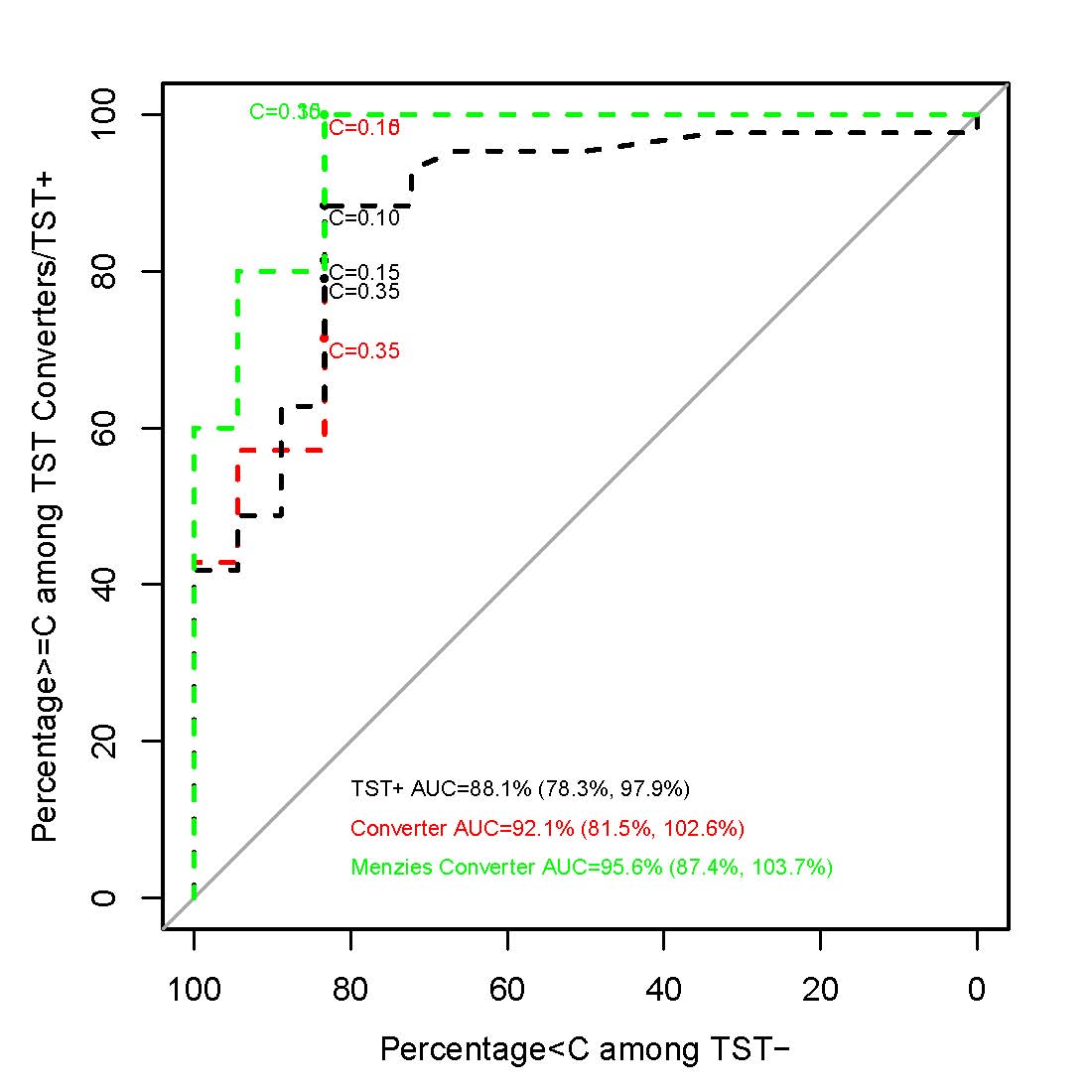


BCG+: 146 TST+, 39 TST Converters (34 if increase<6 mm excluded), 91 TST-

BCG-: 43 TST+, 7 Converters (5 if increase<6 mm excluded), 18 TST-

Tests comparing AUCs for BCG+ vs BCG- for TST+ p=0.49, Converter p=0.007, and Menzies Converter p=0.001.
